# The Clinical Predictors of Malignancy in the Prostate Gland and Their Correlation With Prostate-Specific Antigen (PSA) Levels

**DOI:** 10.7759/cureus.63548

**Published:** 2024-06-30

**Authors:** Aparna Mishra, Sunil Kumar Mahto, Manoj K Paswan, Satyabrata Patra, Aditi Kashyap, Tripti Ashu

**Affiliations:** 1 Pathology, Mahatma Gandhi Memorial Medical College and Hospital, Jamshedpur, IND; 2 Pathology, Rajendra Institute of Medical Sciences, Ranchi, IND

**Keywords:** histopathological analysis, gleason’s grading, prostate cancer, benign prostatic hyperplasia, psa, prostate-specific antigen, prostate gland

## Abstract

Background and objective

The prostate gland, which plays a crucial role in the male reproductive system, has a complex structure and function. Prostate enlargement, often benign but occasionally malignant, poses significant health concerns, particularly in aging populations. Prostate-specific antigen (PSA) serves as a vital biomarker, reflecting changes in prostate architecture and aiding diagnostic stratification. Elevated PSA levels correlate with prostate pathology and standard grading systems such as Gleason grading help guide treatment decisions. This study aimed to investigate the correlation between prostate enlargement, PSA levels, and Gleason grades, particularly within the Indian context.

Materials and methods

This study was conducted over one and a half years at the Department of Pathology, Rajendra Institute of Medical Sciences, Ranchi, and involved 100 cases of clinically enlarged prostates. Clinical data, including age, symptoms, and relevant features, were collected, and histopathological analysis was performed on biopsy specimens. Statistical analysis was conducted using Microsoft Excel and SPSS Statistics version 20.0 (IBM Corp., Armonk, NY).

Results

Our study identified possible links between several factors and prostate conditions. Non-vegetarian diets showed a potential association with increased adenocarcinoma prevalence (p = 0.179). Urinary symptoms like hesitancy, incomplete voiding, retention, frequency, and urgency were significantly more common in men with adenocarcinoma (p<0.05). Additionally, bone pain and abnormal digital rectal examination (DRE) findings strongly correlated with adenocarcinoma (p<0.001). As expected, age showed a positive correlation with prostate weight and PSA levels (p<0.01). Interestingly, bone pain was associated with a lower likelihood of other prostate symptoms (p = 0.023).

Conclusions

Our findings provide key insights into the clinical factors associated with prostate pathology and highlight the need for a comprehensive approach to diagnosis in these patients, integrating clinical evaluation and histopathological assessment.

## Introduction

The prostate gland, a vital component of the male reproductive system, is characterized by its compound tubuloalveolar structure. Situated below the urinary bladder and anterior to the rectum, it encircles the urethra, playing a key role in seminal fluid secretion. This pear-shaped organ, typically weighing around 20 grams in young males, constitutes a minute fraction of the body's weight, yet its functions are indispensable. Composed of both glandular and non-glandular elements tightly enveloped within a capsule, the prostate is anatomically distinguished into four zones: the peripheral, central, transition, and anterior fibromuscular stroma.

Pathological conditions, especially prostate enlargement, mostly afflict the elderly population. While most enlargements are benign, a subset manifests malignancy [1]. The etiology of prostate enlargement is often linked to androgenic factors, besides multifactorial determinants like age, race, and environmental factors [2]. Prostate-specific antigen (PSA), a serine protease primarily synthesized in prostate epithelial cells, serves as a pivotal biomarker. This 34,000-dalton protein, akin to kallikrein, has a serum half-life of 2.2-3.5 days, retaining stability at -20 °C for long periods [3]. Perturbations in prostate architecture, as seen in benign prostatic hyperplasia (BPH), prostatitis, or prostate cancer (PCa), cause elevated PSA levels, aiding diagnostic stratification. High PSA levels, typically ranging from 4 to >10 ng/ml, correlate with prostate pathology, with levels >20 ng/ml indicating advanced PCa [4,5]. Age-stratified reference ranges for PSA levels aid clinical interpretation, revealing the physiological variation across different age groups.

Further complexities arise from the distribution of PSA in serum, with the majority bound to proteins, and a fraction existing as free PSA [6]. In PCa, alterations in the free-to-total PSA ratio serve as a diagnostic indicator, with ratios lower than 0.25 suggesting a higher likelihood of cancer [7]. Standardized grading systems, such as Gleason grading, delineate prostate carcinoma based on architectural features, thereby guiding treatment decisions. This study aims to elucidate the correlation between prostate enlargement, PSA levels, and Gleason grades, particularly within the Indian context, thereby augmenting the diagnostic utility of PSA in PCa diagnosis, a domain that remains largely unexplored outside of Western populations. We aimed to investigate the clinical predictors associated with malignancy of the prostate gland, with a specific focus on correlating these predictors with PSA levels.

## Materials and methods

We employed a hospital-based descriptive design for this study, and it was conducted over one and a half years (January 2020 to June 2021) at the Department of Pathology, Rajendra Institute of Medical Sciences, Ranchi, and involved 100 cases. The inclusion criteria were as follows: patients aged more than 40 years diagnosed with enlarged prostate either by ultrasonography or digital rectal examination (DRE) and having PSA levels above 4 ng/ml. Patients under 40 years of age and those receiving anti-androgens, alpha-blockers, or chemotherapy were excluded.

Specimens obtained from prostate biopsy, transurethral resection of the prostate (TURP), and radical prostatectomy were selected based on the inclusion criteria for histopathological assessment. Informed consent was obtained from each patient, and in cases where the patient was unable to provide consent, consent was acquired from their parents or guardians. Clinical data including age, presenting symptoms, and relevant clinical features were documented from case records, which were mainly subjective in nature, along with macroscopic examination findings of the specimens.

All data including religion, residence, diet, poor stream, hesitancy, terminal dribbling, incomplete voiding, retention, nocturia, frequency, urgency, bone pain, and DRE were meticulously recorded in a structured proforma to maintain accuracy and consistency. A significance level of p≤0.05 was set for statistical analysis. Laboratory procedures involved fixation of the specimens in 10% formalin solution, followed by processing with an automated tissue processor, paraffin embedding, and sectioning at 3-5 microns using a microtome. Sections were stained with hematoxylin and eosin for histopathological analysis.

Statistical analysis was performed using Microsoft Excel and SPSS Statistics version 20.0 (IBM Corp., Armonk, NY). Numerical variables were summarized as mean and standard deviation (SD), while categorical variables were presented as counts and percentages. Logistic regression was applied to assess significant symptoms.

## Results

Among the variables analyzed in our study, dietary habits, urinary symptoms (including hesitancy, incomplete voiding, retention, frequency, and urgency, at the time of presentation), bone pain, and DRE findings demonstrated significant associations with the prevalence of BPH versus adenocarcinoma. Urinary symptoms such as hesitancy (p = 0.001), incomplete voiding (p = 0.045), retention (p = 0.037), frequency (p = 0.023), and urgency (p = 0.066) were significantly associated with adenocarcinoma diagnosis. Additionally, bone pain (p = 0.000) and abnormal DRE findings (p = 0.000) were strongly correlated with adenocarcinoma (Table 1).

**Table 1:** Correlation Analysis of Clinical Variables With Histopathological Diagnoses of Prostate Conditions BPH: Benign Prostatic Hyperplasia; DRE: Digital Rectal Examination

Sl. No.	Variables	Categories	Diagnosis				Prevalence
			BPH		Adenocarcinoma		
			N	%	N	%	
1	Religion	Hindu	59	70.20%	9	56.20%	0.272
		Muslim	25	29.80%	7	43.80%	
2	Residence	Urban	27	32.10%	3	18.80%	0.284
		Rural	57	67.90%	13	81.20%	
3	Diet	Vegetarian	24	28.60%	2	12.50%	0.179
		Non-vegetarian	60	71.40%	14	87.50%	
4	Poor Stream	Present	59	29.80%	13	18.80%	0.369
		Absent	25	70.20%	3	81.20%	
5	Hesitancy	Present	31	36.90%	13	81.20%	0.001
		Absent	53	63.10%	3	18.80%	
6	Terminal Dribbling	Present	46	54.80%	5	31.20%	0.085
		Absent	38	45.20%	11	68.80%	
7	Incomplete Voiding	Present	54	64.30%	6	37.50%	0.045
		Absent	30	35.70%	10	62.50%	
8	Retention	Present	50	59.50%	5	31.20%	0.037
		Absent	34	40.50%	11	68.80%	
9	Nocturia	Present	64	76.20%	15	93.80%	0.114
		Absent	20	23.80%	1	6.20%	
10	Frequency	Present	23	27.40%	9	56.20%	0.023
		Absent	61	72.60%	7	43.80%	
11	Urgency	Present	27	32.10%	9	56.20%	0.066
		Absent	57	67.90%	7	43.80%	
12	Bone Pain	Present	83	98.80%	9	56.20%	0
		Absent	1	1.20%	7	43.80%	
13	DRE	Present	62	73.80%	0	0.00%	0
		Absent	22	26.20%	16	100.00%	

The age distribution in our study was as follows: 51-60 years: 16.0%; 61-70 years: 47.0%; 71-80 years: 30.0%; and 81-90 years: 7.0%. The cohort's mean age was 68.2200 ± 8.3588 years (Table 2).

**Table 2:** Age Distribution of the Study Participants Mean Age: 68.2200 ± 8.3588 Years

Age Group, Years	N (%)
51-60	16 (16.0%)
61-70	47 (47.0%)
71-80	30 (30.0%)
81-90	7 (7.0%)

The correlation analysis revealed significant associations in terms of age, prostate weight, and PSA values in the studied population. Specifically, age exhibited a moderately positive correlation with both weight (r = 0.576, p<0.01) and PSA values (r = 0.436, p<0.01). Moreover, prostate weight (measured by weighing scale before grossing) demonstrated a strong positive correlation with PSA values (r = 0.584, p<0.01). These findings suggest that age, prostate weight, and PSA values are interrelated factors, highlighting the potential importance of considering these variables collectively in clinical assessments and interventions related to prostate health (Table 3).

**Table 3:** Correlation Analysis of Age, Prostate Weight, and PSA Value *Correlation is significant at the 0.01 level (2-tailed) PSA: Prostate-Specific Antigen

Variables		Age, Years	Prostate Wt., gm	PSA Value, ng/ml
Age, Years	Pearson Correlation	1	0.576^*^	0.436^*^
	Sig. (2-tailed)		0.000	0.000
	N	100	100	100
Prostate Wt., gm	Pearson Correlation	0.576^*^	1	0.584^*^
	Sig. (2-tailed)	0.000		0.000
	N	100	100	100
PSA Value, ng/ml	Pearson Correlation	0.436^*^	0.584^*^	1
	Sig. (2-tailed)	0.000	0.000	
	N	100	100	100

The regression analysis indicated that bone pain was significantly associated with a lower likelihood of prostate health-related symptoms (p = 0.023). However, hesitancy, incomplete voiding, retention, and frequency did not show statistically significant associations with prostate health symptoms (p>0.05). The relationship between DRE findings and prostate health symptoms was inconclusive due to the high p-value (p = 0.997) (Table 4).

**Table 4:** Logistic Regression Analysis of Clinical Variables as Predictors of Prostate Malignancy Constant Odds Ratio: 1.684 Variable(s) entered in step 1^a^: Hesitancy, Incomplete Voiding, Retention, Frequency, Bone Pain, DRE DRE: Digital Rectal Examination

Variables		N (%)	Odds Ratio	95% Confidence Interval	
				Lower	Upper
Step 1^a^	Hesitancy	57 (57%)	5.146	0.671	39.457
	Incomplete Voiding	38 (38%)	1.806	0.252	12.957
	Retention	44 (44%)	0.783	0.112	5.463
	Frequency	67 (67%)	1.732	0.291	10.321
	Bone Pain	8 (8%)	0.056	0.005	0.670

## Discussion

This study examined the histopathological features of enlarged prostate glands and their association with serum PSA levels. In their study involving 277 males, Okuja et al. reported a median serum PSA level of 1 (95% CI: 1-2) ng/ml, with most (78.3%) subjects having levels ≤4 ng/ml [8]. The median sonographic prostate volume (PV) was 26 (95% CI: 26-29) ml, with 56.0% showing PV between 25 and 50 ml. PSA levels and PV increased with age, ranging from 0.9 ng/ml and 22 ml in the 30-39 year group to 7 ng/ml and 38 ml in the 60-69 year group. PSA correlated weakly with PV (ρ = 0.27, p<0.0001). Prostatic nodules were present in 47% of participants, with 77% being benign.

Bohnen et al. excluded participants with PCa during baseline and follow-ups (n = 142) [9] and found that higher PSA ranges (>4.0 ng/ml) were more indicative of PV >40 or 50 cc. Multiple regression analysis showed minimal additional information from DRE and age-PSA interaction for PV prediction. PSA alone effectively detected enlarged prostates (>30 cc), with slight improvement when combined with age, DRE, and age-PSA interaction, though clinically modest. Mor et al. excluded patients with specific urinary conditions [10]. The mean age at presentation was 64.4 years, with 34% classified as severe as per the International Prostate Symptom Score (IPSS) grading. Significant correlations were found between post-void residual urine (PVRU) and prostate volume. Acute urinary retention occurred in 16% of cases, and 2% had bladder stones. Benign prostatic enlargement, IPSS score, and post-void residual urine correlated weakly with increasing age. BPH mostly affects elderly individuals, peaking in the fifth and sixth decades of life and correlating with inguinal hernia and acute urinary retention (16%). However, patient age showed no correlation with IPSS score severity.

Ilic et al. collaborated with a parallel guideline committee (BMJ Rapid Recommendation) to inform their systematic review's design and interpretation, focusing on patient-important outcomes [11]. They used a random effects model for pooled incidence rate ratios (IRR) and conducted subgroup analyses based on various factors like age, screening frequency, family history, ethnicity, and socioeconomic level, along with a sensitivity analysis for bias risk. Evidence quality was evaluated using the GRADE approach. Five randomized controlled trials involving 721,718 men were included, with variations in screening frequency, PSA thresholds for biopsy, and bias risk. Overall, screening likely had no effect on all-cause mortality (IRR: 0.99, 95% CI: 0.98-1.01; moderate certainty) and may not affect prostate-specific mortality (IRR: 0.96, 95% CI: 0.85-1.08; low certainty). Godbole et al. emphasized the prevalence of prostatic adenocarcinoma in men over 50, with PSA levels aiding early diagnosis [12]. Their study aimed to correlate histopathological findings with preoperative PSA levels. They analyzed biopsy specimens histopathologically and compared PSA levels with diagnoses.

Freedland et al. studied the relationship between BMI, PSA, and prostate weight in 1,414 men undergoing RP [13]. They found that increasing BMI correlated with increased prostate weight, but only in men younger than 63 years (p<0.001). However, there was no significant association between BMI and prostate weight in men aged 63 years or older (p = 0.44). Younger men with a BMI of 30-34.9 kg/m^2 ^had higher adjusted prostate weights than those with a BMI <25. There was no significant association between BMI and preoperative PSA levels (p = 0.70). Hence, obesity was linked to larger prostate size in men undergoing RP, particularly younger individuals. Dhangar et al. assessed PSA values and their correlation with prostatic biopsy results in patients with prostatic symptoms [14], aiming to understand the association between raised PSA levels in non-carcinoma prostate patients, timing of prostate carcinoma diagnosis in India, and treatment options for newly diagnosed cases. Their study involved 500 patients with lower urinary tract symptoms and prostatic enlargement and found a mean PSA value of 8.5 ng/ml in BPH patients, indicating higher levels in Indian patients due to prostatitis. Most prostate carcinoma cases were diagnosed at advanced stages with metastases.

The mean age in our study was 68.22 years, which aligns with the findings of the study conducted by Mor et al. [10] and Reddy et al. [15]. Reddy V et al. (2019) conducted a prospective study of 36 patients referred to the Department of Radio-Diagnosis at Yenepoya Medical College Hospital, Mangalore, for TRUS-guided biopsy [15]. Biopsies were performed using the sextant core biopsy technique, and samples were evaluated histopathologically. Results were analyzed based on frequency and percentage, revealing a higher incidence of prostatic diseases in older age groups, with most patients demonstrating grade II prostatic enlargement. Our study showed advancing age, nodular prostate, and raised PSA to be significant predictors of malignancy. These findings are in agreement with the study conducted by Mathaiyan et al. [16]

Our study encountered certain limitations. The relatively small sample size of 100 participants restricts the generalizability of the results to larger populations. Additionally, the fact that the study was conducted at a single tertiary care hospital may introduce selection bias. Finally, the coronavirus disease 2019 (COVID-19) pandemic and related lockdown measures during the study period undoubtedly impacted participant recruitment and study execution.

## Conclusions

Our study identified key clinical factors associated with prostate pathology. Urinary symptoms such as hesitancy, incomplete voiding, and frequency, alongside dietary habits, showed associations with adenocarcinoma prevalence. Logistic regression analysis highlighted hesitancy, incomplete voiding, and bone pain as potential predictors of malignancy. Additionally, abnormal findings in DRE were strongly associated with adenocarcinoma. These findings emphasize the importance of a comprehensive approach to diagnosis, integrating clinical evaluation and histopathological assessment. Further research is needed to validate these findings and enhance diagnostic strategies for prostate conditions.
